# Selective sex‐based differences in the association between angiogenic and Alzheimer’s Disease biomarkers in a preliminary cohort of rrAD participants

**DOI:** 10.1002/alz.093284

**Published:** 2025-01-09

**Authors:** Jenny Lutshumba, Donna M. Wilcock, Tiffany Lee, David C Zhu, Norman Scheel, Dwight C German, Rong Zhang, Amanda Trout, Ann M Stowe

**Affiliations:** ^1^ University of Kentucky, Lexington, KY USA; ^2^ Indiana University School of Medicine, Stark Neurosciences Research Institute, Department of Neurology, Indianapolis, IN USA; ^3^ Sanders‐Brown Center on Aging, Lexington, KY USA; ^4^ Michigan State University, East Lansing, MI USA; ^5^ UT Southwestern Medical Center, Dallas, TX USA

## Abstract

**Background:**

Vascular contributions to cognitive impairment and dementia (VCID) and Alzheimer’s disease (AD) are the two most common forms of dementia, with overlapping risk factors including cardiovascular risk factors such as hypertension and dyslipidemia. The etiology of both VCID and AD shows sex‐based differences, as well as sex‐based differences in cardiovascular risk factors. However, how sex differences influence AD and angiogenic biomarkers in older adults who have high cardiovascular risk factors is not known.

**Method:**

AD and angiogenic biomarkers for VCID were measured from the plasma of a subgroup (n=96) of participants from the ‘risk reduction for Alzheimer’s disease’ (rrAD) two‐year clinical trial (NCT02913664; completed Jan. 2022; n=513 total participants). rrAD participants had a family history of dementia or subjective memory complaints, hypertension, and dyslipidemia. The subgroup in the study included 31 males and 65 females (aged: 71‐85). Baseline values for AD biomarkers: Total tau, pTau181, Aβ40, and Aβ42, angiogenic biomarkers: Tie‐2, VEGF‐A, VEGF‐C, VEGFR1, and VCID biomarkers: bFGF, VEGF‐D, PlGF were analyzed using Meso Scale Discovery (MSD). Nonparametric analysis evaluated the sex differences in biomarkers, linear regression evaluated the relationship between AD biomarkers and angiogenic biomarkers in both sexes.

**Result:**

We found sex‐based differences in AD biomarkers such that females had a higher expression in Total tau and Total tau/Aβ42 (p=0.0021, p=0.0003, respectively) while pTau181 was higher in males (p=0.0216). pTau181/ Aβ42 and Aβ42/40 ratios showed no sex differences, nor did baseline angiogenic biomarkers. There was, however, a selective sex difference in the association between angiogenic and AD biomarkers. In females, Total tau is associated with VEGF‐D (R^2^= 0.0746, p=0.0277), and Tau/Aβ42 is associated with VEGF‐A and VEGFR‐1(R^2^= 0.0747, p=0.0275; R^2^= 0.0973, p=0.0114, respectively). In males, pTau181 and pTau181/Aβ42 are associated with VEGF‐D (R^2^= 0.2637, p=0.0031; R^2^= 0.1799, p=0.0174, respectively), Aβ42/Aβ40 is associated with VEGF‐C (R^2^= 0.1491, p=0.0319), and Tau/Aβ42 is associated with bFGF (R^2^= 0.1458, p=0.0340).

**Conclusion:**

There is a selective sex‐based difference in plasma AD biomarkers and their association with angiogenic biomarkers in this preliminary cohort of older adults with high risks of developing Alzheimer’s disease and related dementias.

